# The Effects of a Short Self-Assembling Peptide on the Physical and Biological Properties of Biopolymer Hydrogels

**DOI:** 10.3390/pharmaceutics13101602

**Published:** 2021-10-02

**Authors:** Sumit Chowdhuri, Moumita Ghosh, Lihi Adler-Abramovich, Debapratim Das

**Affiliations:** 1 Department of Chemistry, Indian Institute of Technology Guwahati, North Guwahati, Assam 781039, India; sumit176122040@iitg.ac.in; 2 Department of Oral Biology, The Goldschleger School of Dental Medicine, Sackler Faculty of Medicine, Tel Aviv University, Tel Aviv 6997801, Israel; moumita.g@technoindiaeducation.com; 3 The Center for Nanoscience and Nanotechnology, Tel Aviv University, Tel Aviv 6997801, Israel; 4 The Center for the Physics and Chemistry of Living Systems, Tel Aviv University, Tel Aviv 6997801, Israel; 5 Department of Chemistry, Techno India University, EM-4, EM Block, Sector V, Bidhannagar, Kolkata, West Bengal 700091, India

**Keywords:** composite hydrogel, peptide, hyaluronic acid, bone cell growth

## Abstract

Hydrogel scaffolds have attracted much interest in the last few years for applications in the field of bone and cartilage tissue engineering. These scaffolds serve as a convenient three-dimensional structure on which cells can grow while sensing the native environment. Natural polymer-based hydrogels are an interesting choice for such purposes, but they lack the required mechanical properties. In contrast, composite hydrogels formed by biopolymers and short peptide hydrogelators possess mechanical characteristics suitable for osteogenesis. Here, we describe how combining the short peptide hydrogelator, Pyrene-Lysine-Cysteine (PyKC), with other biopolymers, can produce materials that are suitable for tissue engineering purposes. The presence of PyKC considerably enhances the strength and water content of the composite hydrogels, and confers thixotropic behavior. The hyaluronic acid-PyKC composite hydrogels were shown to be biocompatible, with the ability to support osteogenesis, since MC3 T3-E1 osteoblast progenitor cells grown on the materials displayed matrix calcification and osteogenic differentiation. The osteogenesis results and the injectability of these composite hydrogels hold promise for their future utilization in tissue engineering.

## 1. Introduction

Scaffolds that mimic the natural state of tissue by providing a three-dimensional environment that is biocompatible, non-immunogenic, and acts as an artificial extracellular matrix (ECM), are essential for tissue engineering applications. A variety of materials have been used for such purposes to date, and a combination of inorganic or organic materials with natural polymers are currently in routine use for tissue engineering [1,2,3,4,5,6,7,8,9], where they provide the mechanical and structural support necessary for cell growth. In recent years, much attention has been focused on biodegradable and injectable hydrogels as promising candidates for matrix and scaffolds in tissue engineering [10,11,12], and natural polysaccharide-based hydrogels including chitosan (Cht), hyaluronic acid (HA), and alginate (Alg) are in common use [10,11,13,14,15,16,17]. These materials fulfil the basic criteria of bio-compatibility, hydrophilicity, and capacity for high water storage in their long entangled network, which mimics the natural extracellular matrix and allows cells to adhere and differentiate. However, these biopolymer-based hydrogels suffer from inferior mechanical properties, which make them weaker than the natural ECM and thus they cannot be implanted [6,13,18].

The mechanical properties of such biopolymers can be enhanced and made suitable for tissue engineering by preparing composite hydrogels [19]. However, it is important to ensure that any additives do not compromise the biocompatibility and biodegradability while enhancing the usability of these bio-polymeric hydrogels for bone tissue engineering applications [19,20]. Peptide-based soft materials appear to be a favorable choice as their material properties can be modified by modifying the assembly process, which in turn permits modulation of cellular functionality and tissue morphogenesis [21,22,23,24,25,26,27]. In addition, peptides can self-assemble in aqueous medium to form fibrous network-like structures that can immobilize water molecules through cohesive forces, thus generating self-supporting hydrogels. The advantages of these hydrogels are: (a) they are made of natural amino acids and are generally biocompatible and biodegradable, (b) the extent and proportions of different noncovalent interactions can be readily modified to yield desirable mechanical properties, (c) specific functional groups can be easily incorporated into the sequence to generate smart, stimuli-responsive materials [24]. The mechanical and functional properties can be further tailored by combining two or more gelator molecules to form composite hydrogels [28,29,30]. Co-assembly of two building blocks has previously been used to prepare hybrid materials with physical properties and biocompatibility superior to those displayed by the individual building blocks [25,29,31,32,33].

**Scheme 1:**
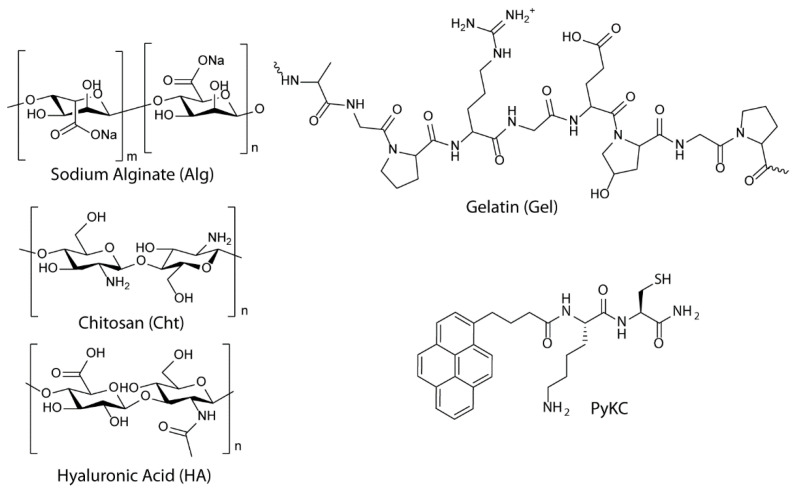
Chemical structures of different biopolymers and PyKC used in this study to construct composite hydrogels.

In this context, we have recently described a tripeptide, PyKC (Scheme 1), which forms a very tightly knitted network of thin fibers in the hydrogel state [34,35]. The glutathione responsive supramolecular PyKC hydrogel remains insoluble in water and other water miscible organic medium, as well as buffers of different pH (3–11), and human serum. In addition, the hydrogel displays extreme confinement properties with a highly restricted exchange of solvents or solutes to and from the gel. As an extension of these findings, we have recently reported another series of similar peptides that have a KC unit at the C-terminal, and whose hydrogels support excellent cell-proliferation in the hydrogel network [36].

Based on these results, we predict that PyKC could be an excellent candidate for a member of a two-component composite hydrogel with biopolymers. The tendency of PyKC to form very tightly woven network has the potential to enhance the mechanical properties, introduce thixotropic/injectable capabilities, and make the resultant composite hydrogels an attractive material for bone tissue engineering. Here, we describe the effects of combining PyKC with four different biopolymers on the resultant hydrogel properties, and particularly on the mechanical behavior. Our results indicate that very small percentage doping with PyKC results in significant improvements in the composite hydrogels. 

Based on the overall properties of these materials, the composite hydrogel prepared by the combination of HA and PyKC was further tested for bone cell proliferation and differentiation and the results indicate that the material represents an excellent candidate for supporting osteogenic differentiation.

## 2. Materials and Methods

### 2.1. General Information and Materials

All the protected amino acids, coupling agents, and resin were procured from GL Biochem, Shanghai, China. Gelatin, sodium alginate, Proteinase K (from Tritirachium albumin), and Minimum Essential Medium Eagle (αMEM), were purchased from Sigma Aldrich (St. Louis, MI, USA). Sodium hyaluronate and chitosan were procured from TCI Chemicals, Japan. Triethylsilane (TES), trifluoroacetic acid (TFA), *N*,*N*-diisopropylethylamine (DIPEA), HPLC-grade dimethylformamide (DMF), and acetonitrile (ACN) were obtained from Spectrochem, Mumbai, India and Fisher Scientific, India. Milli-Q water with a conductivity of less than 2 μS cm^−1^ was used for all sample preparations. Electrospray ionization mass spectrometry (ESIMS) was performed with a Q-Tof-Micro Quadrupole mass spectrometer (Micromass), and data were analyzed using the built-in software (MassHunter Workstation). MALDI analyses were performed with a Daltonics—autoflex™ speed MALDI-TOF instrument (Bruker, Billerica, MA, USA)

### 2.2. Synthesis

PyKC was synthesized following Scheme S1 (Electronic Supplementary Information). The synthetic processes and characterization data are provided in the ESI.

### 2.3. Preparation of the Hydrogel

PyKC stock solution (2 wt %) was prepared in Tris buffer (20 mM, pH 8) and was used immediately to avoid any dimerization before the composite hydrogel preparation. Hyaluronic acid (HA) and sodium alginate (Alg) stock solutions of 20 wt % were prepared by overnight stirring or shaking in ultrapure water. Chitosan (Cht) stock solution (20 wt %) was prepared in 0.1 M acetic acid solution by overnight stirring. For Gelatin (Gel), a readymade 2% solution in water available from Sigma was used directly. For composite hydrogel preparation, two components were mixed in 1:1 ratio by volume (to attain a final concentration of 10 wt % for HA, Alg, and Cht while the concentration of PyKC remained 1 wt %). The mixing was followed by vortexing (30 s) and 24 h incubation at room temperature. The gel composite hydrogel was prepared by the same protocol of mixing, while the final concentration of gel was maintained at 1 wt %. Hydrogels composed of pure biopolymers were prepared by following the same protocols with the required concentrations of biopolymers in the final solution before the 24 h incubation at room temperature to form the hydrogels. It should be noted that Alg and Gel failed to form hydrogels at 10 and 1 wt % concentrations respectively.

### 2.4. Field Emission Scanning Electron Microscopy (FESEM)

Small aliquots (5 μL) of 24 h matured composite and pure biopolymer hydrogels were cast on silicon wafers and dried under ambient conditions for 24 h. Images were recorded by a Gemini SEM 300 (Sigma Zeiss; FESEM) instrument. 

### 2.5. Swelling Property

The pure and composite biopolymer hydrogels were freeze dried individually and the weights (*W_d_*) were recorded before the dried disc shaped materials were immersed in a large excess of water at room temperature for a various periods of time up to 24 h. The water-swollen gels were then centrifuged, removed from the bulk water, and weighed (*W_s_*). The measurements were repeated three times to obtain the mean value, and the swelling ratio (*SR*) was calculated following Equation (1).

(1) $$SR=\frac{W_{s}-W_{d}}{W_{d}}\times 100\%$$

### 2.6. Stability of Hydrogels toward Proteolytic Digestion

The enzymatic degradation of the hydrogels was carried out by using proteinase K. Briefly, a small portion of each hydrogel sample was placed in a 1.5 mL centrifuge tube and incubated with 1 mL of proteinase K solution (3 units/mL) in HEPES buffer (20 mM, pH 7.4). To maintain enzymatic activity, the buffer solution was replaced every 24 h. After a predetermined time, the samples were removed from the solution, excess surface water was gently blotted with tissue paper, and the gels were weighed. The experiments were performed in triplicate to obtain the mean values. The percentage degradation was calculated from the ratio of the final weight to the original weight of the hydrogels.

### 2.7. Stability of Hydrogel in Cell Culture Medium

The composite hydrogel, HA/PyKC was prepared in a glass vial, to which alpha-minimum essential medium (α-MEM) supplemented with 10% fetal calf serum, 100 U/mL penicillin, and 100 U/mL streptomycin was added on top of the hydrogel. The vial was incubated at 37 °C, and photographs were taken at different time points. 

### 2.8. Rheology

The rheological measurements of the pure biopolymer and composite hydrogels were carried out on an Anton Paar MCR 102 rheometer equipped with a 20 mm parallel plate (with 0.3 mm zero gap) measuring system at 25 °C. For the oscillatory tests, an amplitude strain sweep was carried out at frequency of 1 Hz with deformation ranging from 0.01 to 1000%. The frequency sweep from 0.1 to 100 rad/s was made at a controlled deformation (selected from the range of linear viscoelasticity (LVE) determined through amplitude sweep measurements). Cyclic dynamic strain sweep experiments were performed at a constant angular frequency of 1 rad s^−1^ while altering the applied strain from 0.1 to 1000%. According to this protocol, a higher strain (γ = 1000%) and a lower strain (γ = 0.1%) are applied to the gel alternatively over a period of five successive cycles. For temperature dependent rheological experiments, amplitude sweep and frequency sweep experiments were performed at room temperature (25 °C), physiological temperature (37 °C), and at 50 °C. For temperature sweep experiments, samples were first allowed to equilibrate on the rheometer at 25 °C for 3 min, before the temperature was raised from 25 °C to 50 °C at 1 °C/min at a constant frequency of 1 Hz and constant amplitude of 10%, and the viscosity values were recorded. In order to measure the shear stress versus shear rate, the samples were subjected to three stages of hysteresis cycles. In the first stage, the shear rate was increased linearly from 5 s^−1^ to 110 s^−1^, and was then maintained at 110 s^−1^ (second stage). In the final stage, the shear rate was decreased linearly from 110 s^−1^ to 5 s^−1^ and the shear stress was measured accordingly.

### 2.9. Measurement of ESI-MS of the Composite Hydrogel Samples

Small portions of the 24 h matured composite hydrogels in a mixture of water and DMSO were sonicated until a clear solution was obtained. DMSO was used as the hydrogel of PyKC was found to be DMSO soluble. The solutions were then filtered through 0.2 μm syringe filters and analyzed by ESI-MS.

### 2.10. Cell Viability on the Composite Hydrogel

Murine MC3 T3-E1 preosteoblast cells were cultured in α-MEM supplemented with 10% fetal calf serum, 100 U/mL penicillin, and 100 U/mL streptomycin in a Petri dish at 37 °C under a humidified atmosphere in an incubator containing 5% CO_2_. Hydrogels formed in 96-well plate were washed several times with culture medium over 2 days, followed by UV sterilization for 30 min. Cells were then seeded on the hydrogels and incubated at 37 °C under a humidified atmosphere containing 5% CO_2_. The cell viability of the non-differentiating cells was assessed by the MTT assay 3 days after seeding. For this purpose, MTT stock solution (5 mg/mL) was prepared in phosphate buffer saline (PBS), and 20 µL solution was added to each well followed by a 4 h incubation. After this time, the MTT reduced adduct (formazan) formed was extracted by the addition of 100 µL DMSO to each well and shaking for 20 min. The absorbance of each well was then recorded using a Tecan Spark plate reader (at 570 nm) with a background correction at 680 nm. 

Cell viability on HA-PyKC composite hydrogels was also assessed qualitatively by Live/Dead staining at the same time points as the MTT assays. Hydrogels were prepared in a 24-well plate and rinsed repeatedly with culture medium, followed by UV sterilization, for 2 days. Cells were then seeded on the hydrogels and cultured for 3 days. A solution containing fluorescein diacetate (6.6 µg/mL, for live cells) and propidium iodide (5 µg/mL, for dead cells) was added and the labeled cells were immediately viewed using a Nikon Eclipse Ti fluorescent microscope. Images were captured by a Zyla scMOS camera using a Nikon Intensilight C-HGFI fluorescent lamp.

### 2.11. Alkaline Phosphatase (alp) Activity

To measure intracellular ALP activity, hydrogels were formed in a 96-well plate and rinsed repeatedly with culture medium for 2 days, followed by 30 min UV sterilization before seeding of cells. MC3 T3-E1 preosteoblasts (3000 cells) were seeded on the prewashed hydrogels as before and the culture medium was supplemented with differentiation medium (ascorbic acid and β-glycerophosphate) every 2 days for a period of 14 days. After this time, the hydrogels were stained by addition of 100 µL ALP substrate solution containing 4-methylumbelliferyl phosphate (4-MUP), and incubated for 30 min in the dark. The fluorescence was then measured (λ_ex_ = 360 nm and λ_em_ = 440 nm). Each reading was normalized to the appropriate cell count for the hydrogel.

### 2.12. Mineralization Assay

The Alizarin red staining assay, which quantifies the amount of mineralization from bone nodule formation, was used to determine the extent of matrix mineralization of MC3 T3-E1 preosteoblasts on the hydrogels. The hydrogels were formed in a 24-well plate and washed repeatedly with culture medium for 2 days, followed by UV sterilization for 30 min before seeding of cells, for 2 days. MC3 T3-E1 preosteoblasts (3000 cells) were seeded on the prewashed hydrogels and cultured as before with supplementation of differentiation medium every 2 days for a period of 14 days. After this time, the cells were stained with Alizarin red, and imaged optically after washing off excess dye. The following day, the deposited calcium was dissolved in 2:1 MeOH/AcOH buffer, and the samples were shaken for 45 min to ensure complete dissolution of the calcified matrix. The percentage of calcification was quantified by reading the absorbance of the solution at 405 nm. Each reading was normalized to the cell count in the relevant hydrogel.

### 2.13. Statistical Analysis

All experiments were repeated three times with triple repeats of each sample each time, and the results are expressed as mean ± standard error of the mean. The statistical analysis of differences between groups of cytocompatibility assays and pre-vs post-differentiation were performed with a significance of *p* < 0.01 of the three repeats of each sample in triplicate experiments determined by Mann–Whitney U test using the Microsoft Excel program. 

## 3. Results

### 3.1. Preparation of Polymer/PyKC Composite Hydrogels

PyKC was combined with four different biopolymers: chitosan (Cht), sodium alginate (Alg), gelatin (Gel), and hyaluronic acid (HA, Scheme 1). These polymers were selected because they are all capable of forming hydrogels under different experimental conditions and have provided promising results when used as artificial ECM, either in combination with other materials or as the sole component [37]. Our idea was to incorporate PyKC molecules into these polymers in order to generate composite hydrogels with properties superior to those of the parent materials. Initially, polymer/PyKC composite hydrogels were prepared by mixing the two components 1:1 by volume to produce materials with 1 wt % polymer and 1 wt % PyKC. However, since this ratio resulted in very weak hydrogels (data not shown), the polymer–PyKC ratio was changed to 10:1 (wt %). Unless otherwise mentioned, all studies were performed with gels prepared using these proportions. Notably, in the case of gelatin, it was difficult to construct such a composite hydrogel (with 10 wt % of Gel) owing to the poor solubility of gelatin in aqueous medium. Thus, a 1:1 (wt %) Gel/PyKC ratio was used for all further studies of this material. Hereafter, the composites are denoted as, polymer short name (Cht, Alg, Gel, or HA)/PyKC. No hydrogel could be formed for Alg and Gel at 10 and 1 wt % concentrations respectively in the absence of PyKC. 

### 3.2. Characterization of Polymer/Pykc Composite Hydrogels

A series of characterization studies were performed to examine and compare the properties of the composite hydrogels to those of hydrogels prepared with the polymers alone or only with PyKC. PyKC forms disulfide linked dimers in neutral to basic conditions due to the presence of cysteine residue in the sequence [34]. These dimers have been previously shown to be crucial for the hydrogelation of PyKC [34]. ESI-MS analyses of 24 h matured samples of HA/PyKC and Gel/PyKC composite hydrogels showed the presence of a peak corresponding to the PyKC dimer (Figures S1 and S2). In contrast, even after careful investigations, no mass corresponding to the PyKC monomer could be detected. Notably, both monomeric as well as dimeric masses were detected in samples of the other two composites, (Figures S3 and S4). Analytical HPLC analyses of HA/PyKC and Gel/PyKC confirmed the lack of PyKC monomers, although incomplete dimerization was observed for the other two composites (Figure S5). The extents of dimerization for Cht and Alg composites were estimated to be 65 and 70% respectively, and these values did not improve even after incubation for 7 days. The gel melting temperatures (*T_g_*) of all the composites were measured by the ball dropping method (Table S1). The results indicated that HA/PyKC and Gel/PyKC hydrogels were stable up to 90 °C with no signs of melting observed at this temperature. In contrast, the melting temperatures of Alg/PyKC and Cht/PyKC composites were 80 °C and 72 °C respectively. Importantly, the *T*_g_ of PyKC is reported to be 75 °C [34].

**Figure 1:**
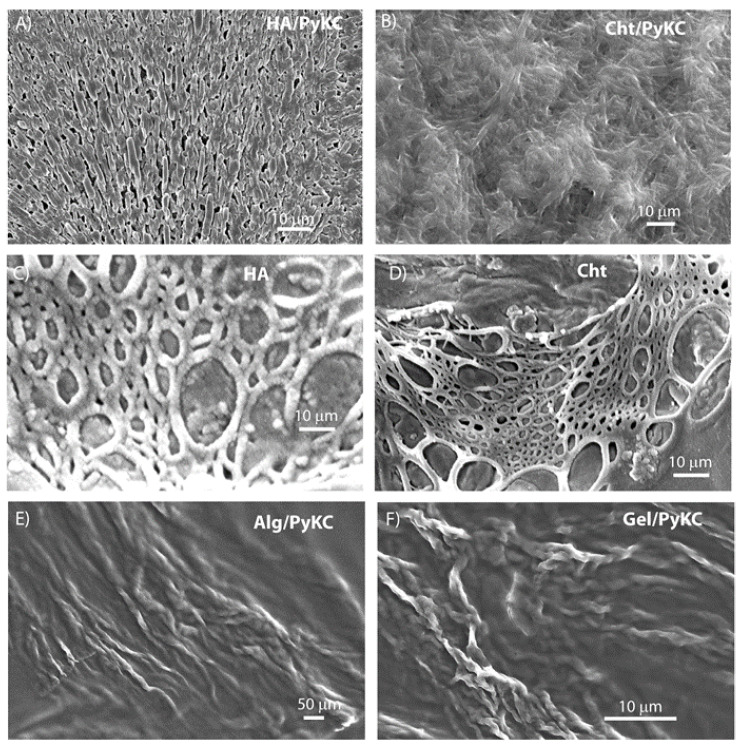
FESEM images of different hydrogels. (**A**) HA/PyKC (10:1 wt %); (**B**) Cht/PyKC (10:1 wt %); (**C**) HA (10 wt %); (**D**) Cht (10 wt %); (**E**) Alg/PyKC (10:1 wt %); and (**F**) Gel/PyKC (1:1 wt %).

The morphologies of the composite hydrogels were evaluated by FESEM. Well-arranged brick like structures were observed for HA/PyKC, (Figure 1A), but the Cht/PyKC composite hydrogel was composed of a long entangled fibrillar network with the fibers measuring several micrometers (Figure 1B). As can be seen in Figure 1C,D, these morphologies are significantly different from those of hydrogels composed of the relevant polymers alone at this concentration. No fibrillar network was observed for Alg/PyKC and Gel/PyKC, which instead displayed a dense bundle like morphology.

Next, the swelling behavior of these composite hydrogels was studied [38]. As can be seen in Figure S6, the highest swelling ratio (Table S1) was noted for Gel/PyKC with a value of 32.50, while Alg/PyKC had the lowest (12.41), and the Cht and HA composites, had values of 16.08 and 14.81, respectively. Importantly, the presence of PyKC increased the swelling ratio when compared to the respective parent polymer hydrogels. This might be due to enhanced water retention in a 3 D hydrogel structure containing PyKC, and may be attributed to the tightly knitted network previously seen for the PyKC hydrogel [34].

### 3.3. Rheological Characterization of Polymer/PyKC Composite Hydrogels

**Figure 2:**
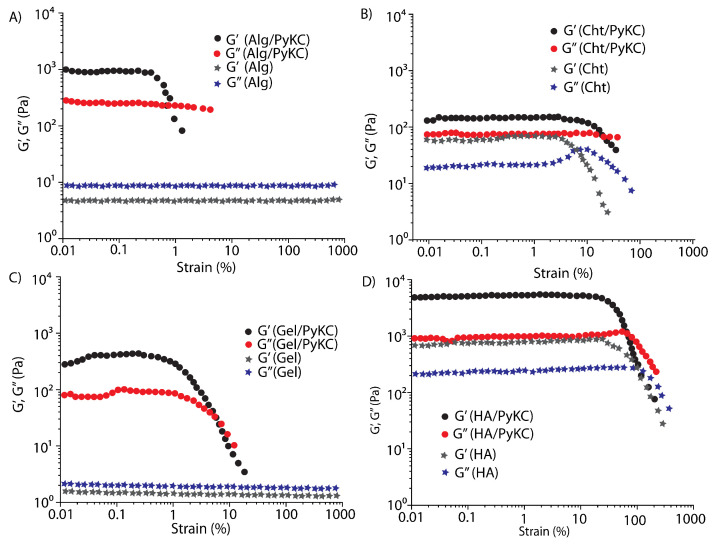
Changes in storage and loss moduli as a function of shear strain (amplitude sweep at a frequency of 1 rad·s^−1^) for (**A**) Alg and Alg/PyKC, (**B**) Cht and Cht/PyKC, (**C**) Gel and Gel/PyKC, and (**D**) HA and HA/PyKC hydrogels measured at room temperature.

Rheological analyses were performed in order to examine the hydrogel formation kinetics and mechanical properties. For this purpose, the hydrogels were subjected to 0.01–1000% strain at a constant frequency. As expected, both Alg and Gel samples displayed higher G” (loss modulus) values in the amplitude sweep experiments than the corresponding G’ (storage modulus), reflecting the liquid character of the materials that fail to form any hydrogel (Figure 2) [39]. Rheological strain sweep analysis revealed a wide linear viscoelastic region of up to ~10% strain for Cht/PyKC and Gel/PyKC hydrogels, while the Alg/PyKC hydrogel displayed a linear viscoelastic region up to 1% strain. Interestingly, the HA/PyKC hydrogel displayed strain sustainability up to 100%. The G’ results obtained from the frequency sweep experiments (0.01–1000 rad/s) on the prepared hydrogels indicated that the HA/PyKC hydrogel is the strongest (~5000 Pa), followed by Gel/PyKC (~4000 Pa), Alg/PyKC (~2000 Pa), and Cht/PyKC (~100 Pa, Figure S7). These results are interesting as although Gel and Alg on their own were unable to form a hydrogel under similar experimental conditions, the addition of only 1% PyKC, resulted in a moderately strong hydrogel. Notably, in all cases, the presence of PyKC, enhanced the gel strength considerably. As a good gelator, PyKC probably provides the driving force for the supramolecular organization with these polymers via H-bonding, thus producing a composite hydrogel with high mechanical rigidity. The hydrophilic/lipophilic balance (HLB) [40] of Cht/PyKC hydrogel is probably beyond the range that allows the material to form a rigid gel.

**Figure 3:**
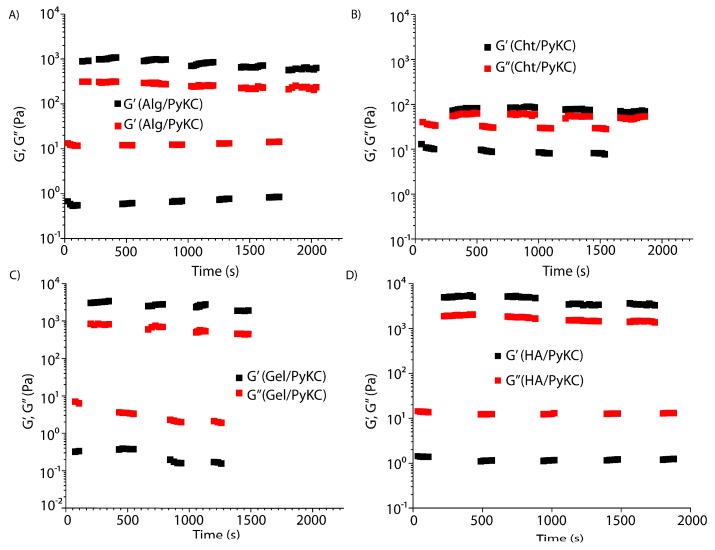
Thixotropic behavior of the composite hydrogels (**A**) Alg/PyKC, (**B**) Cht/PyKC, (**C**) Gel/PyKC, and (**D**) HA/PyKC as reflected by the continuous step strain measurements at room temperature.

The standard rheological analyses were followed by thixotropy measurements. Thixotropy of hydrogels is an important property that can predict the potential for bio-medical applications, and in particular for localized drug delivery and 3 D cell-proliferation [39,41,42]. A thixotropic hydrogel exhibits thinning in response to a shear strain and then returns to the original gel state when the strain is removed [43]. All the composite hydrogels described here demonstrated good thixotropic characteristics, although the hydrogels formed by 10 wt % HA and Alg were not thixotropic. Figure 2 shows that all the composite hydrogels exhibit yield strain and transform into quasi-liquids, which is indicative of the start of deformation. Time dependent strain sweeps were executed at a fixed angular frequency (1 rad·s^−1^), and by alternating the applied strains (Figure 3). The results indicate that the viscoelastic properties of all the composite hydrogels were lost at higher strain, but were restored when lower strain was applied, thereby confirming that all the hydrogels are thixotropic. The injectability of the composite gels was tested by preparing the hydrogels in a syringe and then injecting them into water. In all cases, the composite hydrogels could easily be injected (Figure S8).

It is clear that the presence of PyKC significantly alters the aggregation characteristics of the polymers. The presence of PyKC-dimers in the composites, and the observation that Alg and Gel required the presence of PyKC to form hydrogels, provide evidence for the critical role of PyKC in the hydrogel formation of these composites. Moreover, the presence of PyKC not only enhances the mechanical strength of the composites but also increases the water uptake, as well as introducing thixotropic behavior. 

**Figure 4:**
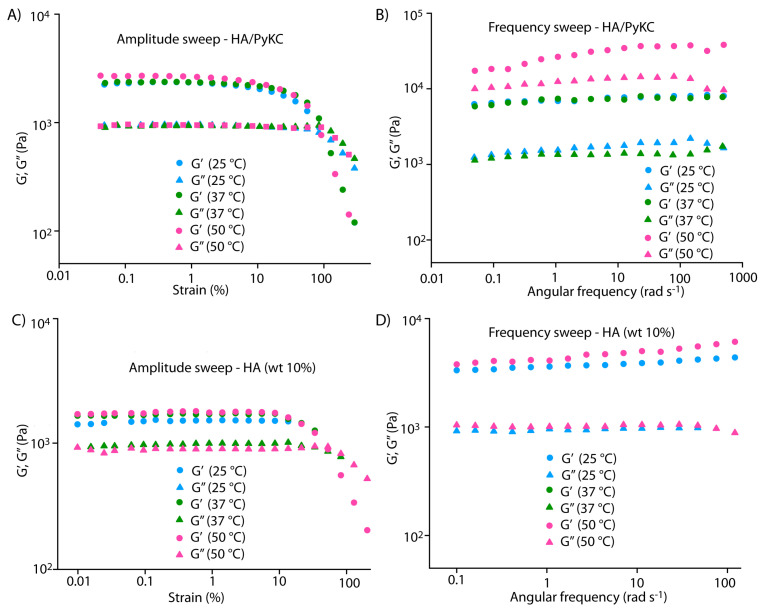
Temperature dependent amplitude sweep (**A**,**C**) and frequency sweep (**B**,**D**) of HA/PyKC and HA hydrogels respectively.

As the next stage, we conducted temperature dependent rheological analyses of the HA/PyKC and Alg/PyKC systems. For this purpose, amplitude and frequency sweep experiments were performed at three different temperatures (25, 37, and 50 °C). As can be seen in Figure 4 and Figure S9, the HA/PyKC hydrogel displayed strain sustainability up to 100% at all three temperatures. The G’ values from the frequency sweep experiments indicated that the gels become stronger with increasing temperature and retain their stability. In contrast, the strain sustainability of the Alg/PyKC hydrogel was only maintained to 2% strain (Figure S9). The results of the temperature sweep experiments indicated that both the HA/PyKC and 10 % HA hydrogels resist dissolution at higher temperatures. Interestingly, the hysteresis loop obtained for the HA/PyKC hydrogel clearly indicates that the hydrogel was not damaged at a higher shear rate, and it returned to the initial condition when the shear rate was lowered (Figure S10). In addition, although the 10% HA hydrogel was damaged at higher shear rates, the structure reformed after rest and eventually returned to the initial state. 

### 3.4. Biocompatability of the Polymer/PyKC Composite Hydrogels

**Figure 5:**
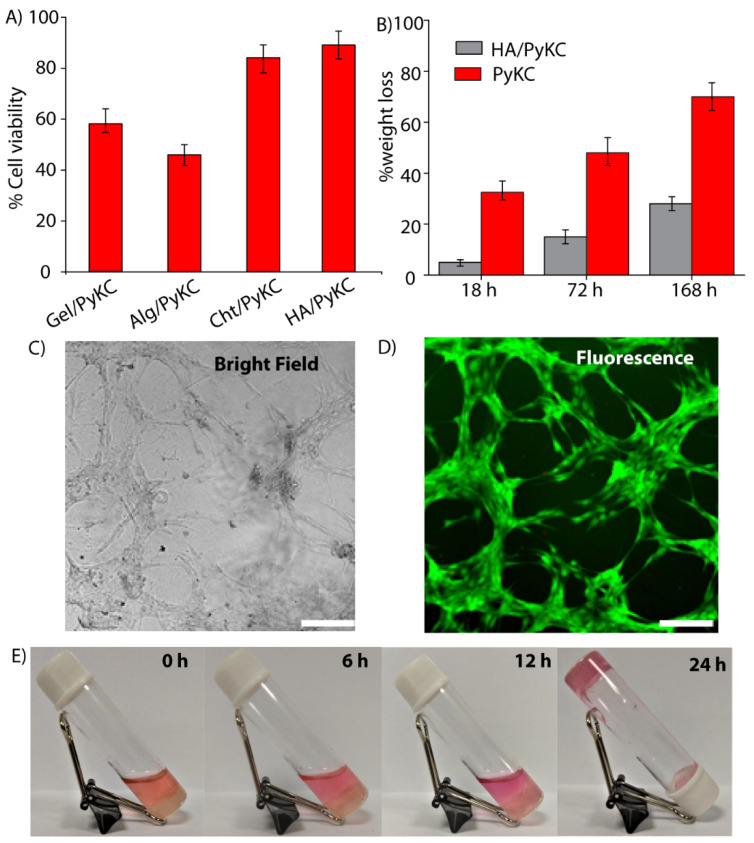
Cytocompatibility and stability of the composite hydrogels. (**A**) Cytotoxicity profile of different composite hydrogels toward MC3 T3-E1 preosteoblast cells after 3 days (mean values ± SD; n = 9). Statistics: Mann–Whitney U test *p* < 0.01). (**B**) Stability of the HA/PyKC and PyKC hydrogels against proteinase K digestion. (**C**) Bright field image of MC3 T3-E1 cells on Hyaluronic acid/PyKC composite hydrogel. Scale bar = 100 μm (**D**) Fluorescence spectroscopic image of live MC3 T3-E1 cells on Hyaluronic acid/PyKC composite hydrogel. Scale bar = 100 μm. (**E**) Photographs of HA/PyKC hydrogel incubated in αMEM for different time intervals demonstrating the stability of the composite hydrogel in the culture medium.

To evaluate the potential of the polymer/PyKC composite hydrogels as a scaffold for tissue engineering, their biocompatibility was assessed by in vitro cell culture experiments. Unfortunately, since the Cht/PyKC hydrogel was too weak for the cell regeneration studies, we decided to proceed only with the other three polymer/PyKC composite hydrogels. MC3 T3-E1 preosteoblast cells were seeded on prewashed Alg/PyKC, HA/PyKC, and Gel/PyKC composite hydrogels. The viability of the MC3 T3-E1 cells grown for 3 days on the HA/PyKC hydrogel was 85–90%, as evaluated by MTT assay, indicating high bio-compatibility of the hydrogel. Similar results were obtained for Gel/PyKC and Alg/PyKC hydrogels with viability values of 60–65% and 37–40% respectively (Figure 5A). 

Among all the composite hydrogels tested, HA/PyKC demonstrated superior characters in terms of mechanical properties and biocompatibility. Thus all further studies were carried out using the HA/PyKC composite hydrogel. Since bio-stability of any hydrogel is a prerequisite for bio-medical applications, we evaluated the stability of the HA/PyKC hydrogel against proteolytic digestion [11]. Figure 5B shows that the resistance of the composite hydrogel to proteinase K digestion was higher than that of the PyKC hydrogel. As can be seen (Figure 5B), even after 7 days incubation, only 28% of the composite hydrogel was lost compared to ~70% of the PyKC hydrogel. These results that the composite hydrogel is very resistant to enzymatic digestion and also provide evidence that the two composite components mutually enhance the quality of the hydrogel for bio-medical applications.

The stability of the HA/PyKC hydrogel in the culture medium was further tested by incubating the hydrogel in α-MEM supplemented with 10% fetal calf serum, 100 U/mL penicillin, and 100 U/mL streptomycin. Photographs of the hydrogel were taken at different time intervals. It is apparent that the culture medium gradually permeated the hydrogel matrix as the color of the hydrogel intensified over time (Figure 5E). However, the composite hydrogel was observed to be quite stable after 24 h of incubation, and inverting the vial, revealed the gel to be intact. Rheological analyses of this hydrogel indicated only a minor decrease in strength (Figure S11). 

### 3.5. Osteogenesis on the HA/PyKC Composite Hydrogel

Bone regeneration on artificial scaffolds is an important area of research [44], and the mechanical properties of the HA/PyKC composite hydrogel make it a suitable candidate for an artificial scaffold for bone regeneration [45,46,47]. In order to test this further, we used MC3 T3-E1 pre-osteoblast cells to assess the ability of the scaffold to support pre-osteoblast growth, proliferation, and differentiation. The morphology of the cells on HA/PyKC hydrogel after 3 days were observed by Live/Dead staining which uses a cell membrane dye (fluorescein diacetate, green) to detect live cells, and a DNA stain (propidium iodide, red) to stain dead cells. The results revealed large numbers of green cells with good shape and morphology (Figure 5C,D), and no propidium iodide staining was observed. These results provide further evidence of the viability of MC3T3-E1 cells grown on the HA/PyKC hydrogel.

**Figure 6:**
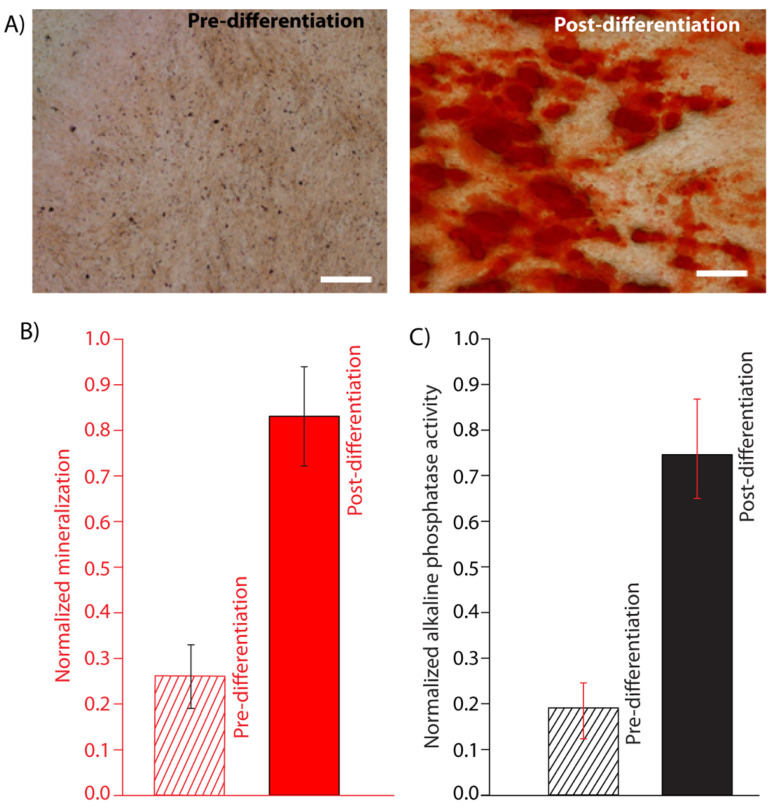
Osteogenic response of MC3 T3-E1 cells on the HA/PyKC composite hydrogel. (**A**) Microscope images of MC3 T3-E1 preosteoblast cells stained with Alizarin red before (pre-differentiation) and after (post-differentiation) 14 days of osteogenic differentiation on HA/PyKC hydrogel. Scale bar = 500 μm. (**B**) Quantification of calcification by Alizarin red staining of MC3 T3-E1 preosteoblasts before and after 14 days of osteogenic differentiation on HA/PyKC hydrogel (mean values ± SD; n = 9). Statistics: Mann–Whitney U test *p* < 0.01). (**C**) Quantification of alkaline phosphatase (ALP) activity of MC3 T3-E1 preosteoblast cells before and after 14 days of osteogenic differentiation on HA/PyKC hydrogel (mean values ± SD; n = 9). Statistics: Mann–Whitney U test *p* < 0.01). Scale bar = 500 μm.

As the next step, we evaluated the extent of differentiation and matrix mineralization of the MC3 T3-E1 cells on the HA/PyKC composite hydrogel by Alizarin red assay. Extracellular calcium deposits are produced by pre-osteoblasts during mineralization. These deposits can be stained by Alizarin red and quantified to determine the extent of mineralization arising from bone nodule formation. The amount can then be normalized to the number of cells. In contrast to the results pre-differentiation, the cellular matrix displayed an intense red stain after 14 days of differentiation on the HA/PyKC hydrogel (Figure 6A). Quantifying the Alizarin red staining, indicated that the intensity was ~4 times higher in the differentiated cells (Figure 6B) than in the pre-differentiated cells. 

In addition, we evaluated the osteogenic differentiation the MC3T3-E1 cells by assaying the alkaline phosphatase (ALP) activity of cells grown on the composite hydrogel, since this serves as a marker of early differentiation. Following 14 days incubation in osteogenic medium, ALP activity in the seeded MC3T3-E1 cells was measured by addition of an ALP substrate, 4-methylumbelliferyl Phosphate (4-MUP). As can be seen in Figure 6C, the ALP activity was ~3.5 times higher in the differentiated than in the pre-differentiated cells. This suggests that the HA/PyKC composite hydrogel is capable of supporting an osteogenic response in the osteoblast progenitor MC3T3-E1 cells. These results provide convincing evidence that the HA/PyKC hydrogel efficiently induces the differentiation and mineralization of MC3T3-E1 preosteoblast cells and thus meets the pre-requisites of a potential bone regeneration scaffold. 

## 4. Conclusions

We have successfully demonstrated the effects of a short peptide, PyKC, on the gelation process and hydrogel properties of different biopolymers. Systematic analyses of the composite hydrogels of PyKC with four different biopolymers demonstrate that the presence of PyKC enhances the water content and mechanical strength of the scaffold and also introduces thixotropic properties. Importantly, all these changes are achieved with very small percentage doping of the peptide. The composite hydrogel of HA/PyKC was found to be the most suitable to serve as a scaffold and is capable of supporting inducing osteogenic differentiation in osteoblast progenitor cells. The high degree of matrix mineralization and ALP activity displayed by the pre-osteoblast cells growing on the composite hydrogel provides evidence for the high potential of the scaffold for future bone tissue engineering applications. Moreover, the thixotropic behavior also introduces the possibility of evaluating this composite hydrogel for use in artificial synovial fluid. 

## Data Availability

Not applicable.

## Supplementary Materials

The following are available online at https://www.mdpi.com/article/10.3390/pharmaceutics13101602/s1, Scheme S1: Schematic route for the synthesis of PyKC. Synthetic procedures, Table S1: Compositions and different properties of the hydrogels studied, Figure S1: ESI-MS of 24 h matured composite hydrogel of Gel/PyKC, Figure S2: ESI-MS of 24 h matured composite hydrogel of HA/PyKC, Figure S3: ESI-MS of 24 h matured composite hydrogel of Cht/PyKC, Figure S4: ESI-MS of 24 h matured composite hydrogel of Alg/PyKC, Figure S5: HPLC traces of 24 h samples of different composite hydrogels showing the extent of monomer to dimer conversion of PyKC, Figure S6: Swelling ratio of different hydrogels, Figure S7: Frequency sweep profile of different hydrogels studied at room temperature, Figure S8: Photographs of all the composite hydrogels (rhodamine loaded) during injection in bulk water showing injectability property, Figure S9: Temperature dependent A) amplitude sweep and B) frequency sweep of Alg/PyKC hydrogel, Figure S10: Hystersis loops of HA/PyKC and HA hydrogels measured at RT, Figure S11: Frequency sweep profile HA/PyKC composite hydrogel before and after 24 h incubation in α-MEM supplemented with 10% fetal calf serum, 100 U/mL penicillin, and 100 U/mL streptomycin.
